# The impact of vascular anastomosis time on early kidney transplant outcomes

**DOI:** 10.1186/2047-1440-2-8

**Published:** 2013-05-15

**Authors:** Karim Marzouk, Joseph Lawen, Ian Alwayn, Bryce A Kiberd

**Affiliations:** 1 Department of Medicine, Dalhousie University, 5082 Dickson Building, Queen Elizabeth Health Sciences-VG site, 5280 University Ave, Halifax NS B3H 1V7, Canada; 2 Department of Urology, Dalhousie University, Rm 294 5th Victoria Building, Queen Elizabeth Health Sciences-VG site, 1276 South Park Street, Halifax NS B3H 2Y9, Canada; 3 Department of Urology, Dalhousie University, Rm 5015 5th Floor Centennial Building, Queen Elizabeth Health Sciences-VG site, 1276 South Park Street, Halifax NS B3H 2Y9, Canada; 4 Department of Surgery, Dalhousie University, Rm 6302 6th Floor Centennial Building, Queen Elizabeth Health Sciences-VG site. 1276 South Park Street, Halifax NS B3H 2Y9, Canada

**Keywords:** Delayed graft function, Warm ischemic time, Cold ischemic time, Hospitalization

## Abstract

**Background:**

Most studies have found cold ischemic time to be an important predictor of delayed graft function in kidney transplantation. Relatively less is known about the warm time associated with vascular anastomosis and early outcomes.

**Methods:**

A retrospective cohort of 298 consecutive solitary deceased donor kidney recipients from January 2006 to August 2012 was analyzed to examine the association between anastomosis time and delayed graft function (need for dialysis) and length of hospital stay.

**Results:**

Delayed graft function (DGF) was observed in 56 patients (18.8%). The median anastomosis time was 30 minutes (interquartile range 24, 45 minutes). Anastomosis time was independently associated with DGF in a multivariable, binary logistic regression analysis (odds Ratio (OR) 1.037 per minute, 95% CI 1.016, 1.057, *P* = 0.001). An anastomosis time >29 minutes was also associated with a 3.5 fold higher (OR 3.5, 95% CI 1.6, 7.3, *P* = 0.001) risk of DGF. Median days in hospital was 9 (interquartile range 7, 14 days). Every 5 minutes of longer anastomosis time (0.20 days per minute, 95% CI 0.13, 0.27, *P* <0.001) was associated with 1 extra day in hospital in a multivariable linear regression model. An anastomosis time >29 minutes was associated with 3.8 (95% CI 1.6, 6.0, *P* <0.001) more days in hospital.

**Conclusion:**

Anastomosis time may be an underappreciated but modifiable variable in dictating use of hospital resources. The impact of anastomosis time on longer term outcomes deserves further study.

## Background

Over the last two decades cold ischemic time (CIT) has been found to be an important independent risk factor for delayed graft function (DGF) in deceased donor kidney transplantation [1-4]. DGF is also associated with inferior graft survival and function [1-3]. All supporting evidence for the adverse events associated with DGF is derived from cohort studies. DFG likely results in longer stays in hospital and additional resource use (dialysis). Surprisingly there are few studies examining the effects of warm ischemic time (WIT) on DGF [5,6]. The importance of WIT has become a hot area of research in Urology especially with greater use of partial nephrectomy for cancer surgery [7,8]. There is also recent information that long WITs may reduce graft survival in live donor kidney transplantation [9].

By definition there are two WITs. The first relates to organ procurement and the second relates to the vascular anastomosis time (AT) in the recipient. The purpose of this study is to examine the second warm time or the AT during the recipient operation and examine whether this contributes to delayed graft function.

## Methods

Consecutive deceased donor organ transplant recipients of solitary kidneys were identified in the Program database from January 2006 until August 2012. Combined organ and living donor transplant procedures were excluded. Data were extracted retrospectively from our electronic health records, which came into existence in January 2006. Approval for this retrospective study was obtained from our institution’s research ethics board.

All patients received induction with either basiliximab or antithymocyte globulin (ATG) and all received preoperative methylprednisolone (500 mg) intravenously. ATG was reserved for those who were repeat transplant recipients, those who received organs from deceased cardiac donors, and those with a calculated panel reactive antibody level >20%. Post-operative patients received oral tacrolimus, mycophenolate, and prednisone. All patients were treated with sulfamethoxazole-trimethoprim for pneumocystis prophylaxis and oral ranitidine for gastrointestinal ulcer prophylaxis. For patients on a proton pump inhibitor pre transplant, this agent was continued in place of the ranitidine. Cytomegalovirus (CMV) prophylaxis with valganciclovir was used in CMV-negative recipients receiving CMV-positive organs and CMV-positive recipients receiving ATG. All patients received subcutaneous heparin for venous thromboembolism prophylaxis.

Data extracted included recipient age, gender, body mass index (BMI), weight, calculated panel reactive antibodies (cPRA), human leukocyte antigen mismatch (HLA MM), cause of end-stage renal disease (ESRD), diabetes mellitus status at transplantation, duration of dialysis pretransplantation, blood transfusion pre transplantation, CIT, donor cause of death, donor age, gender, BMI, weight, terminal creatinine, and donation after circulatory death (DCD) versus neurologic death. Since over 96% of the population was non-black, race was not included in the analysis. AT was defined as the time between the end of the cooling period to successful renal artery anastomosis and perfusion of the donor kidney. In the few patients requiring reoperation the cross-clamped warm time of the second operation was included. The first warm time from the procurement process was not included. This first warm time is generally much shorter than the second [5]. The outcomes were DGF (defined as need for dialysis post transplantation within the first week), days in hospital, and recipient serum creatinine level at day 7.

Data are presented as mean ± standard deviation for continuous variables with a normal distribution and median and interquartile range (IQR) for those variables not distributed normally. Differences in AT between groups with or without delayed graft function were tested by the Kruskal-Wallis test. Variables associated with DGF were examined by binary logistic regression analysis. Variables associated with days in hospital and serum creatinine on day 7 were examined by linear regression analysis. Variables examined for statistical associations with outcomes were recipient age, gender, BMI, weight, cPRA, HLA MM, duration of dialysis, blood transfusion pre transplantation (never versus yes), diabetes mellitus status at transplantation, CIT, donor cause of death (anoxia versus other, and cerebrovascular event versus other), donor age, gender, weight, BMI, terminal serum creatinine, DCD, and AT. Variables that were significant at *P* <0.1 in a univariable analysis were included into the multivariable models. Overall significance was assumed at the *P* = 0.05 level (two-sided). Statistical analysis was performed using IBM SPSS Statistics software version 20.0 (New York, NY, USA).

## Results

**Figure 1:**
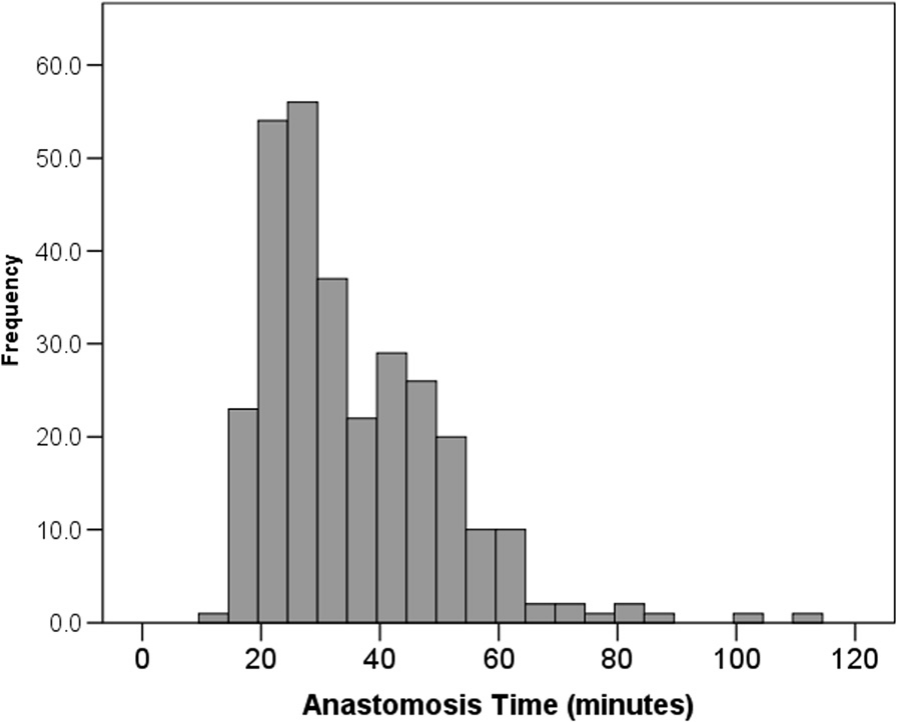
Histogram of anastomosis times.

**Figure 2:**
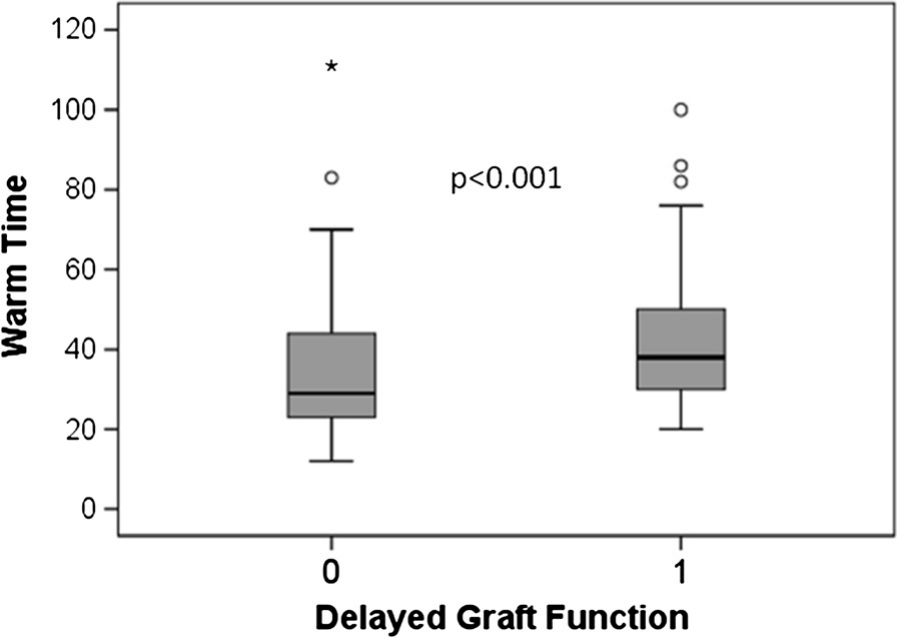
Delayed graft function (1 = yes, 0 = no) and anastomosis (warm) time.

In this retrospective cohort of 307 consecutive deceased donor solitary kidney transplant recipients in the database, ATs were available in 298 subjects. The nine (2.9%) missing subjects were excluded from the analysis. Of the remaining 298 patients, 56 (18.8%) had DGF. Characteristics of the patients are shown in Table 1. Figure 1 shows the distribution of ATs in the study. Since the distribution is significantly skewed the median is the more appropriate descriptor (median 30 minutes, IQR 24, 45 minutes). Figure 2 (box and whisker plot) shows that patients experiencing DGF had significantly longer ATs. AT was independently associated with DGF in the binary logistic regression analysis (odds ratio (OR) 1.037 per minute, 95% CI 1.016, 1.057, *P* = 0.001). Other variables that were significant in the model were DCD (OR 11, 95% CI, 3.3, 34, *P* <0.001), donor BMI (OR 1.06 per kg/m^2^, 95% CI, 1.003, 1.12, *P* = 0.041), and dialysis duration pre transplantation (OR 1.001 per day, 95% CI, 1.000, 1.002, *P* <0.001). Diabetes mellitus, donor sex, and repeat transplants were included in the multivariable model (*P* <0.10 in the univariable model) but were not independently significant. In a separate multivariable model, an AT >29 minutes was associated with an increased risk of DGF (OR 3.5, 95% CI 1.6, 7.3, *P* = 0.001).

**Table 1:** Population descriptors (n = 298)

**Descriptor**	**Value**
Age, years, mean ± SD	51 ± 13
Gender, male, number (%)	181 (60.7%)
Regraft, number (%)	29 (9.0%)
Diabetes mellitus, number (%)	69 (23%)
End-stage renal disease	
Polycystic kidney disease, number (%)	55 (19%)
Glomerulonephritis, number (%)	85 (29%)
Diabetes mellitus, number (%)	50 (17%)
Recipient weight, kg, mean ± SD	78 ± 17
Recipient body mass index, kg/m^2^, mean ± SD	28 ± 5
Blood transfusion, never, number (%)	205 (69%)
Human leukocyte antigen mismatch, mean ± SD	4.1 ± 1.4
Dialysis duration, days, median (IQR)	819 (419, 1462)
Calculated panel reactive antibody, %, median (IQR)	0 (0, 8)
Cold ischemic time, hours, median (IQR)	12 (8, 16)
Anastamosis time, minutes, median (IQR)	30 (24, 45)
Donation after circulatory death, number (%)	16 (5.4%)
Donor cause of death	
Anoxia, number (%)	24 (8%)
Cerebrovascular event, number (%)	189 (62%)
Donor age years, mean ± SD	47 ± 17
Donor male, number (%)	135 (45%)
Donor weight kg, mean ± SD	76 ± 19
Donor body mass index kg/m^2^, mean ± SD	27 ± 6
Donor creatinine μmol/L, mean ± SD	67 ± 31
Delayed graft function, number (%)	56 (18.8%)
Days in hospital, days, median (IQR)	9 (7, 14)
Creatinine day 7, μmol/L, median (IQR)	168 (125, 314)

**Figure 3:**
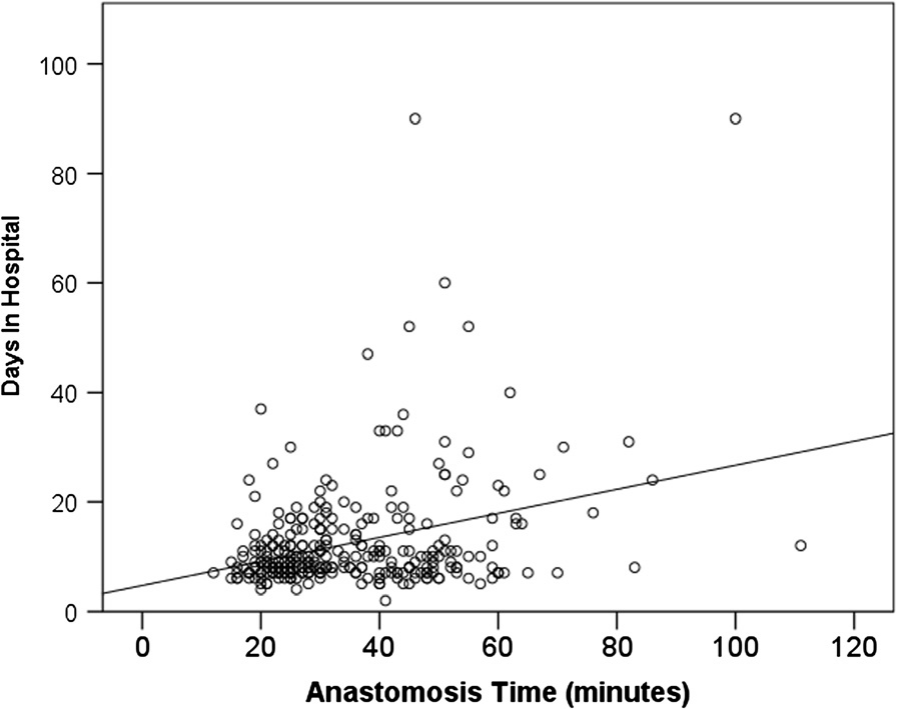
Influence of anastomosis time on days in hospital.

The median days in hospital was 9 (IQR 7, 14 days). Figure 3 shows the relationship between AT and days in hospital (*r* = 0.32, *P* <0.001). AT (B coefficient 0.20 days per minute AT, 95% CI, 0.13, 0.27, *P* <0.001) was associated with longer stays in hospital in the multivariable linear regression analysis model. Therefore every 5 minutes of longer AT was associated with one extra day in hospital. Other significant covariables were DCD (B coefficient 11 days, 95% CI, 7, 16, *P* <0.001), dialysis duration pre transplantation (B coefficient 0.002 days per day, 95% CI, 0.001, 0.003, *P* = 0.001) and donor sex (B coefficient 3.5 days for male, 95% CI, 1.4, 5.6, *P* = 0.001). Recipient age, diabetes mellitus, donor age, and repeat transplants were included in the multivariable model (*P* <0.10 in the univariable model) but were not independently significant. In a separate multivariable model an AT >29 minutes was associated with 3.8 days (95% CI 1.6, 6.0, *P* <0.001) longer in hospital.

The median serum creatinine on day 7 was 168 μmol/L (IQR 125, 314). AT (B coefficient 4.0 μmol/L per minute AT, 95% CI, 2.1, 5.0, *P* <0.001) was associated with higher day 7 creatinine levels. Other significant factors in the multivariable model were donor terminal serum creatinine (B coefficient 1.8 μmol/L per μmol/L, 95% CI, 1.04, 2.5, *P* = 0.001) and DCD (B coefficient 312 μmol/L, 95% CI, 213, 411, *P* <0.001). Recipient sex, recipient weight, diabetes mellitus, and donor BMI were included in the multivariable model (*P* <0.10 in the univariable model) but were not independently significant. An AT >29 minutes was associated with a higher creatinine at day 7 (B coefficient 75 μmol/L, 95% CI, 30, 121, *P* <0.001).

## Discussion

AT may be an underappreciated but modifiable variable in dictating use of hospital resources. There were significant increases in both need for dialysis and length of stay at this center with increased AT. An AT >29 minutes was associated with particularly worse outcomes. The study also shows that the serum creatinine was higher 7 days after transplantation with longer ATs, consistent with slower recovery of kidney function.

Surprisingly few studies have examined WIT as a covariate in studies of delayed graft function or survival. In a normogram to predict DGF, Irish *et al*. identified WIT as a significant variable but the overall effect was small [6]. The reasons why this study demonstrated a greater effect with AT is not clear since most of the variables included in the DGF normogram were also considered in this study. The DGF normogram was examined in one center and found to have limited accuracy [10]. In Grossberg’s study, transplant recipients with and without DGF had DGF normogram scores that were not significantly different. DGF as defined in this paper and by Irish *et a*l. may not be the best predictor of later graft function [6]. Moore *et al*. found the DGF normogram to be less predictive of later kidney function at 1 year than other measures. Creatinine at day 5, and to a similar extent creatinine at day 7 post kidney transplantation were predictive of later function at 1 year [11]. In a single center study from the Netherlands of over 1,000 solitary deceased and live donor kidney recipients transplanted between 1981 and July 2000, neither the first nor second warm time was significantly associated with outcomes [5]. However, a more recent study from the same country in live donor kidney recipients found that prolonged warm times (>45 minutes) were associated with a >3-fold higher risk of graft loss [9]. Most research has focused on CIT, preservation solutions or the use of machine perfusion pumps to avoid reperfusion injury and DGF and these do not directly address the AT [12]. Even the large recent randomized trials such as the international trial on the use of machine pumps to prevent DGF or recent cohort trials examining risk factors for DGF did not include WIT in their analysis as a potential confounder [4,13].

There is a growing body of evidence in the urological literature that WIT in partial nephrectomy is detrimental and there should be no reason why these adverse effects associated with WIT would be irrelevant in kidney transplantation [7,8]. Consistent with a debate in the urologic literature, there may not be a threshold effect and every minute may count [7]. In a recent publication examining the effect of WIT in a cohort undergoing partial nephrectomy of a solitary kidney, it was found that each minute of warm ischemia was associated with a 6% increased risk of acute kidney injury and 4% increased risk of new onset ESRD [14]. Clearly some prolonged times are unavoidable, especially in those with multiple vessels that have significant atherosclerosis or that require re-exploration. Minimizing warm time by back table surgery would also be recommended. Recognizing that pursuing speed should not impact the quality of anastomosis, increasing the awareness of this issue amongst surgeons may be sufficient to improve ATs. It might be necessary to include more simulation and skills laboratory time for trainees to gain adequate expertise prior to performing anastomoses in clinical practice.

There are significant limitations to this study. This was retrospective and single center, and long-term effects on graft or patient survival were not examined because of the low event rates (98% patient and 96% graft survival at one year). Given the growing body of clinical and experimental evidence that acute ischemic kidney injury is associated with later progressive chronic kidney disease in the non-transplant setting, a more detailed analysis of larger registries should be undertaken [15-17]. If an association between greater AT and worse long-term graft survival is observed, then there would be even greater interest in testing early pharmacologic interventions or devising better means to minimize the warm time. A number of strategies have been tested experimentally and in ongoing clinical trials (clinicaltrials.gov) to prevent ischemia-reperfusion injury. In addition to previously studied interventions such as spironolactone in animals, therapies to prevent leukocyte recruitment and inflammation (NCT00248040), cytokine damage (NCT00298168) and the use of small interfering RNAs (NCT00802347) to prevent renal injury may hold promise [17,18].

In summary, this study highlights the importance of the anastomosis time in kidney transplantation on short-term outcomes that may impact long-term outcomes. More significantly this event may be modifiable, or potentially an area and time for other types of intervention.

## Abbreviations

AT: Anastomosis time; ATG: Antithymocyte globulin; BMI: Body mass index; CIT: Cold ischemic time; CMV: Cytomegalovirus; cPRA: Calculated panel reactive antibody; DCD: Donation after circulatory death; DGF: Delayed graft function; DIH: Days in hospital; ESRD: End-stage renal disease; GN: Glomerulonephritis; HLA MM: Human leukocyte antigen mismatch; PCKD: Polycystic kidney disease; OR: Odds ratio; WIT: Warm ischemic time; IQR: Interquartile range.

## Competing interests

None of the authors declare any funding for this study or competing interests. However JL, IA and BK are participating in a contract research study with Quark Pharmaceuticals.

## Authors’ contributions

KM collected data and helped draft the manuscript. JL co-designed the study, and critically appraised the analysis and significance. IA critically appraised the analysis and significance. BK co-designed the study, collected and analyzed data, and assisted in manuscript preparation. All authors read and approved the final manuscript.

## References

[B1] Ojo AO Wolfe RA Held PJ Port FK Schmouder RL Delayed graft function: risk factors and implications for renal allograft survival Transplantation 1997 63 968 974 10.1097/00007890-199704150-00011 9112349

[B2] Lebranchu Y Halimi JM Bock A Chapman J Dussol B Fritsche L Kliem V Oppenheimer F Pohanka E Salvadori M Soergel M Tufveson G MOST International Study Group Delayed graft function: risk factors, consequences and parameters affecting outcome-results from MOST. A multinational observational study Transplant Proc 2005 37 345 347 10.1016/j.transproceed.2004.12.297 15808638

[B3] van der Vliet JA Warlé MC Cheung CL Teerenstra S Hoitsma AJ Influence of prolonged cold ischemia in renal transplantation Clin Transplant 2011 25 E612 E616 10.1111/j.1399-0012.2011.01510.x 21919965

[B4] Kayler LK Srinivas TR Schold JD Influence of CIT-induced DGF on kidney transplant outcomes Am J Transplant 2011 11 2657 2664 10.1111/j.1600-6143.2011.03817.x 22051325

[B5] Roodnat JI Mulder PG Van Riemsdijk IC IJzermans JN van Gelder T Weimar W Ischemia times and donor serum creatinine in relation to renal graft failure Transplantation 2003 75 799 804 10.1097/01.TP.0000056632.00848.8D 12660505

[B6] Irish WD Ilsley JN Schnitzler MA Feng S Brennan DC A risk prediction model for delayed graft function in the current era of deceased donor renal transplantation Am J Transplant 2010 10 2279 2286 10.1111/j.1600-6143.2010.03179.x 20883559

[B7] Amit R Patel AR Eggener SE Warm ischemia less than 30 minutes is not necessarily safe during partial nephrectomy: every minute matters Urol Oncol-Semin O I 2011 2 826 828 10.1016/j.urolonc.2011.02.015 22078406

[B8] Becker F Van Poppel H Hakenberg OW Stief C Gill I Guazzoni G Montorsi F Russo P Stöckle M Assessing the impact of ischaemia time during partial nephrectomy Eur Urol 2009 56 625 634 10.1016/j.eururo.2009.07.016 19656615

[B9] Hellegering J Visser J Kloke HJ D’Ancona FC Hoitsma AJ van der Vliet JA Warlé MC Deleterious influence of prolonged warm ischemia in living donor kidney transplantation Transplant Proc 2012 44 1222 1226 10.1016/j.transproceed.2012.01.118 22663989

[B10] Grossberg JA Reinert SE Monaco AP Morrissey P Utility of a mathematical normogram to predict delayed graft function: a single center experience Transplantation 2006 81 155 159 10.1097/01.tp.0000188621.54448.c8 16436956

[B11] Moore J Ramakrishna S Tan K Cockwell P Eardley K Little AM Rylance P Shivakumar K Suresh V Tomlinson K Ready A Borrows R Identification of the optimal donor quality scoring system and measure of early renal function in kidney transplantation Transplantation 2009 87 578 586 10.1097/TP.0b013e3181949e71 19307797

[B12] Moers C Smits JM Maathuis MH Treckmann J van Gelder F Napieralski BP van Kasterop-Kutz M van der Heide JJ Squifflet JP van Heurn E Kirste GR Rahmel A Leuvenink HG Paul A Pirenne J Ploeg RJ Machine perfusion or cold storage in deceased-donor kidney transplantation N Engl J Med 2009 360 7 19 10.1056/NEJMoa0802289 19118301

[B13] Dikdan GS Mora-Esteves C Koneru B Review of randomized clinical trials of donor management and organ preservation in deceased donors: opportunities and issues Transplantation 2012 94 425 441 10.1097/TP.0b013e3182547537 22892991

[B14] Thompson RH Lane BR Lohse CM Leibovich BC Fergany A Frank I Gill IS Blute ML Campbell SC Every minute counts when the renal hilum is clamped during partial nephrectomy Eur Urol 2010 58 340 345 10.1016/j.eururo.2010.05.047 20825756

[B15] Chawla LS Amdur RL Amodeo S Kimmel PL Palant CE The severity of acute kidney injury predicts progression to chronic kidney disease Kidney Int 2011 79 1361 1369 10.1038/ki.2011.42 21430640 PMC3257034

[B16] Bonventre JV Yang L Cellular pathophysiology of ischemic acute kidney injury J Clin Invest 2011 121 4210 4221 10.1172/JCI45161 22045571 PMC3204829

[B17] Barrera-Chimal J Pérez-Villalva R Rodríguez-Romo R Reyna J Uribe N Gamba G Bobadilla NA Spironolactone prevents chronic kidney disease caused by ischemic acute kidney injury Kidney Int 2013 83 93 103 10.1038/ki.2012.352 23014458

[B18] Molitoris BA Dagher PC Sandoval RM Campos SB Ashush H Fridman E Brafman A Faerman A Atkinson SJ Thompson JD Kalinski H Skaliter R Erlich S Feinstein E siRNA targeted to p53 attenuates ischemic and cisplatin-induced acute kidney injury J Am Soc Nephrol 2009 20 1754 1764 10.1681/ASN.2008111204 19470675 PMC2723992

